# Prolonged Survival of Anaplastic Thyroid Carcinoma: A Case Report and Literature Review

**DOI:** 10.1002/ccr3.71691

**Published:** 2025-12-19

**Authors:** Dereje G. Andargie, Chernet T. Mengistie, Biruk T. Mengistie, Solomon Hunegnaw, Anduamalak Bazezew, Kristin Long, Raj Petersson

**Affiliations:** ^1^ Department of Surgery, College of Medicine and Health Science University of Rwanda Kigali Rwanda; ^2^ School of Medicine, College of Health Sciences Addis Ababa University Addis Ababa Ethiopia; ^3^ School of Medicine, College of Health Sciences Hawassa University Hawassa Ethiopia; ^4^ School of Medicine, College of Health Sciences Bahir Dar University Bahir Dar Ethiopia; ^5^ Department of Surgery, School of Medicine and Public Health University of Wisconsin–Madison Madison Wisconsin USA; ^6^ Department of Otolaryngology – Head and Neck Surgery, School of Medicine Virginia Commonwealth University Richmond Virginia USA

**Keywords:** anaplastic thyroid carcinoma, case report, multi‐modal therapy, neoadjuvant chemotherapy, prolonged survival

## Abstract

Anaplastic thyroid carcinoma (ATC) is a rare, highly aggressive thyroid malignancy that typically progresses rapidly and carries a poor prognosis. Diagnosis is often straightforward when a rapidly enlarging neck mass produces compressive symptoms, but optimal outcomes require urgent multidisciplinary care. We report a 60‐year‐old woman who presented with a 6‐month history of a rapidly growing left anterior neck mass causing progressive hoarseness and dyspnea. Imaging demonstrated a bulky left thyroid lesion with marked tracheal compression, and ultrasound‐guided fine needle aspiration cytology showed undifferentiated pleomorphic tumor cells consistent with ATC. After multidisciplinary review, she received neoadjuvant systemic chemotherapy to achieve cytoreduction, followed by near‐total left thyroidectomy with excision of adherent soft tissue and tracheal decompression; adjuvant systemic therapy was completed postoperatively. Postoperative recovery was uncomplicated apart from transient dysphonia, and final histopathology confirmed ATC. She completed adjuvant systemic therapy and remains in clinical remission, defined by the absence of measurable disease on contrast‐enhanced imaging and sustained clinical stability, 21 months after treatment initiation. While not generalisable, this case suggests that selected patients with locally advanced, nonmetastatic ATC may achieve sustained remission with coordinated multimodal therapy even in resource‐limited settings without access to radiotherapy or targeted agents.

## Key Clinical Message

Aggressive, multidisciplinary management, including neoadjuvant chemotherapy followed by surgical resection and appropriate adjuvant therapy, can produce unexpectedly prolonged survival in selected patients with locally advanced, nonmetastatic anaplastic thyroid carcinoma, even in resource‐limited settings; early multidisciplinary evaluation and individualized treatment planning are essential.

## 1 Introduction

Anaplastic thyroid carcinoma (ATC) is a rare yet highly lethal form of thyroid cancer, representing only about 1%–2% of thyroid malignancies [1, 2]. It predominantly affects elderly patients (median age~60–70 years) with a slight female predominance [3, 4]. ATC is almost invariably advanced at diagnosis; most patients present with a rapidly enlarging neck mass causing dyspnea, dysphagia, or hoarseness [5, 6]. About half of patients already have distant metastases at presentation [6, 7]. ATC tumors are undifferentiated epithelial neoplasms often arising from preexisting differentiated thyroid cancer (papillary or follicular) via a dedifferentiation process [3, 5, 8]. Histologically, ATC shows pleomorphic patterns including spindle cells, giant cells, and squamoid cells with extensive necrosis; fine‐needle aspiration cytology can correctly suggest ATC in many cases (≈84% accuracy) [3]. By definition, ATC behaves as AJCC Stage IV; survival is dismal (median 3–6 months, 1‐year survival < 20%, and 10‐year survival < 5%) [3, 8].

Most clinical guidelines advocate an urgent, multimodal treatment approach to ATC [6]. Whenever feasible, complete surgical resection of the tumor is attempted, often followed by high‐dose external‐beam radiotherapy and systemic therapy [6, 9]. However, surgery is only possible in a minority of patients due to local invasion, and conventional chemotherapy (e.g., doxorubicin ± platinum) has shown low response rates [9, 10]. In recent years, novel targeted and immune therapies have extended options for ATC. For example, up to ~30%–40% of ATCs harbor the BRAF V600E mutation, and dual BRAF/MEK inhibition (dabrafenib plus trametinib) has achieved objective responses in clinical trials [11, 12]. Immune checkpoint inhibitors (e.g., anti‐PD1) are also under investigation [11, 13]. Nevertheless, the overall prognosis remains poor, and there is no consensus “standard of care” proven to cure ATC [5, 9].

This report describes a 60‐year‐old Ethiopian woman with ATC who, contrary to expectation, survived 21 months post‐diagnosis with aggressive combined therapy. We present this case to highlight that prolonged survival is possible in selected patients with locally advanced, nonmetastatic ATC treated with coordinated multimodal therapy despite limited access to radiotherapy and targeted agents, and to compare this outcome with published reports from resource‐limited settings.

## 2 Clinical History/Examination

A 60‐year‐old woman from northwest Ethiopia presented to our Hospital with a six‐month history of a progressively enlarging anterior neck mass associated with increasing hoarseness of voice and exertional breathlessness that progressed to orthopnea over the preceding month. She denied fever, weight loss, dysphagia to solids or liquids, or symptoms suggestive of thyroid dysfunction. Her past medical history was unremarkable, and she was not on regular medications. There was no personal or family history of thyroid disease or radiation exposure.

On examination, the patient was comfortable at rest but reported dyspnea on minimal exertion. Vital signs were within normal limits. Inspection revealed a visible left‐sided anterior neck swelling; palpation confirmed a solitary, well‐defined mass in the left anterolateral thyroid region measuring approximately 9 × 6 cm. The mass was hard, non‐tender, moved with swallowing, and had irregular margins. There was no obvious overlying skin change. Cervical lymph nodes were not clinically enlarged. Cardiorespiratory and neurological examinations were otherwise unremarkable.

### 2.1 Differential Diagnosis, Investigations, and Treatment

**FIGURE 1:**
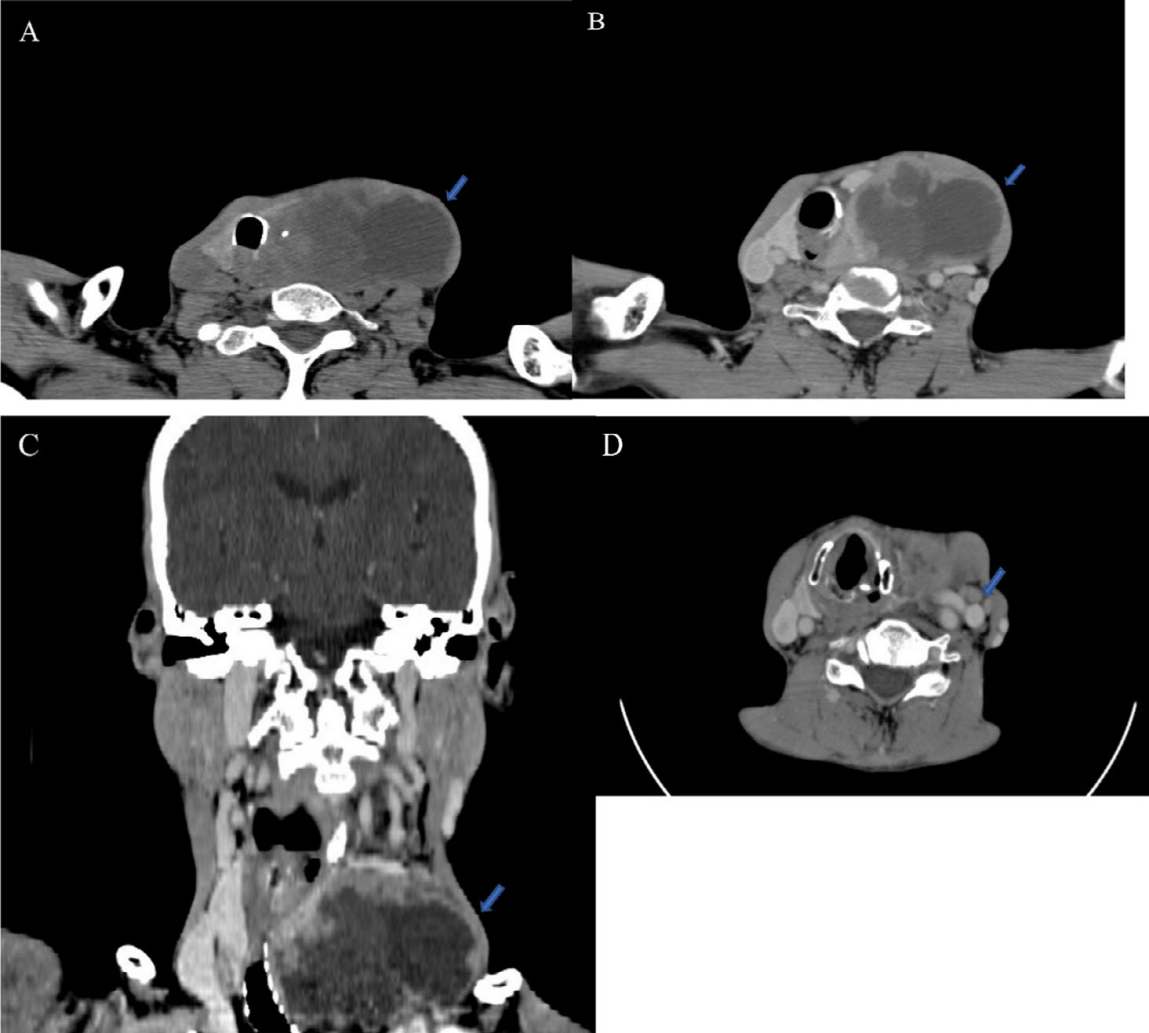
(A–D) Contrast‐enhanced CT of the neck. (A) Non‐contrast axial image showing a complex mass in the left lobe of the thyroid (arrow). (B) Post‐contrast axial image demonstrating heterogeneous enhancement of the mass (arrow). (C) Post‐contrast coronal image confirming complex enhancement (arrow). (D) Axial image showing enlarged left cervical lymphadenopathy (arrow).

Baseline laboratory investigations showed euthyroid biochemistry and no major metabolic derangement. Thyroid function tests were: TSH 1.8 mIU/L, free T4 14.2 pmol/L. Complete blood count and basic metabolic panel were within institutional reference ranges. These results did not alter the diagnostic impression of ATC. Diagnostic workup also included neck ultrasonography, contrast‐enhanced computed tomography (CT) of the neck, and ultrasound‐guided fine needle aspiration cytology (FNAC). Ultrasound showed a large heterogeneous left thyroid lesion with internal solid components and mass effect on adjacent structures. Contrast CT demonstrated a bulky left thyroid mass causing marked tracheal compression and deviation without gross intrathoracic extension (Figure 1A–D).

**FIGURE 2:**
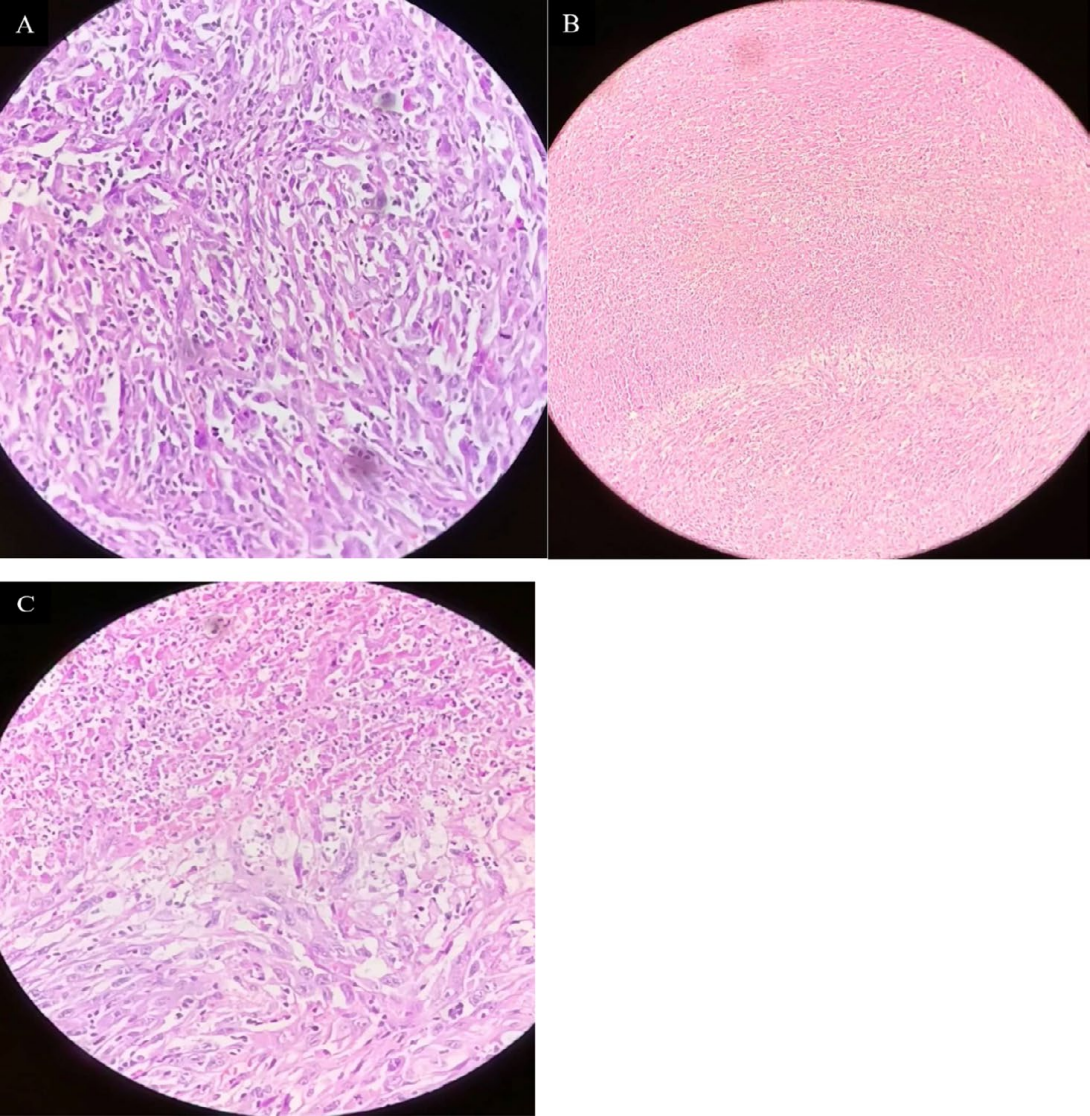
(A–C) Histopathological images of fine needle aspiration cytology from the thyroid mass showing features consistent with anaplastic thyroid carcinoma. (A) Pleomorphic spindle and giant tumor cells with marked nuclear atypia (H&E, ×40). (B) Sheets of undifferentiated tumor cells with areas of necrosis (H&E, ×10). (C) High‐power view demonstrating abundant mitotic activity and multinucleated giant cells within a necrotic background (H&E, ×40).

FNAC yielded highly cellular smears composed of markedly pleomorphic, spindle, and giant tumor cells with abundant mitoses and necrotic background, cytomorphology interpreted as anaplastic (undifferentiated) thyroid carcinoma (Figure 2A–C). Immunohistochemistry was not performed due to local resource constraints; the diagnosis was made on characteristic cytomorphology and confirmatory resection histopathology. Clinical staging was cT4aN0M0 (locally advanced, nonmetastatic), corresponding to AJCC stage IVB, based on tracheal invasion with no radiologic evidence of regional nodal enlargement or distant metastases.

**FIGURE 3:**
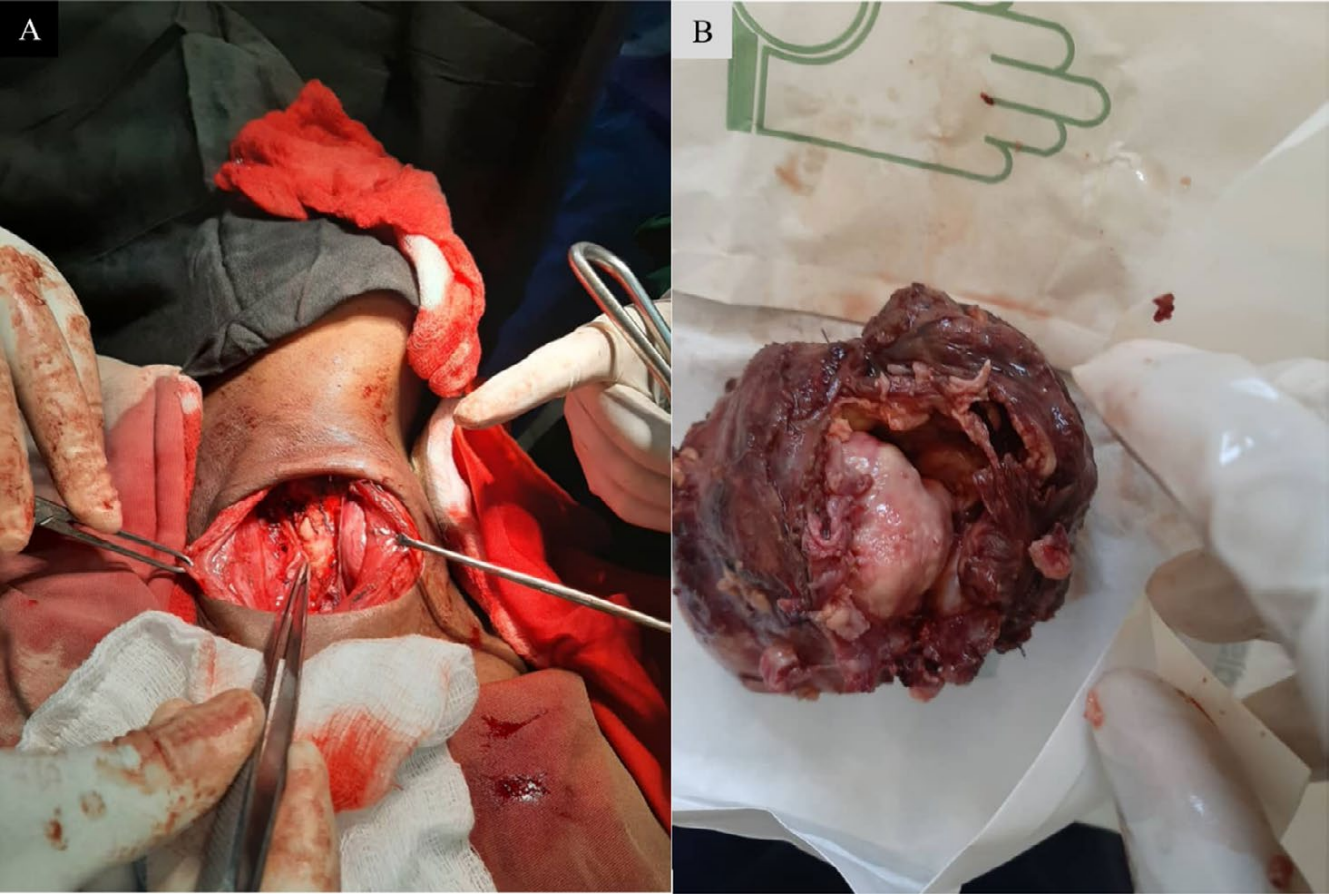
(A–B) Intraoperative view and corresponding gross specimen of the bulky left thyroid mass. (A) Intraoperative photograph through a transverse (Kocher) incision showing exposure of a large left anterolateral neck mass occupying the left thyroid region; the tumor is densely adherent to the strap muscles and produces mass effect on the trachea (retractors and forceps in the field). The recurrent laryngeal nerve was identified and preserved where it was not grossly invaded. (B) Resected specimen after near‐total left thyroidectomy demonstrating a bulky, irregular tumor with a heterogeneous cut surface and areas of solid tumor, hemorrhage, and necrosis. Final histopathology confirmed anaplastic (undifferentiated) thyroid carcinoma.

Following multidisciplinary tumor board discussion, the patient received neoadjuvant systemic chemotherapy aimed at tumor cytoreduction and symptom relief. She received paclitaxel 80 mg/m^2^ IV weekly for 6 cycles, which produced measurable clinical and radiologic cytoreduction, permitting safer resection. After reassessment, the patient underwent a standard transverse cervical (Kocher) incision and subplatysmal flap elevation. Intraoperatively, the mass was largely confined to the left thyroid lobe, densely adherent to the overlying strap muscles and compressing the trachea; the recurrent laryngeal nerve was identified and preserved where not grossly invaded (Figure 3A,B). A near‐total left thyroidectomy with excision of involved adjacent soft tissue and isthmusectomy was performed with careful hemostasis; tracheal decompression (shaving of compressive tissue) was achieved without formal tracheal resection. Surgical margins were focally positive microscopically (R1) at the deep margin related to tracheal surface adherence; three regional lymph nodes were sampled and were negative for metastatic disease (0/3). A closed suction drain was left in situ.

Postoperative recovery was uneventful; the patient had transient dysphonia that gradually improved, and no immediate airway compromise or hypocalcaemia requiring intervention. Final histopathology confirmed the diagnosis of ATC. The patient completed adjuvant systemic therapy with weekly paclitaxel 80 mg/m^2^ for an additional 6 cycles. Radiotherapy and targeted therapies were not given because they were unavailable locally.

### 2.2 Outcome and Follow‐Up

At 21 months after treatment initiation, the patient remains in clinical remission. Posttreatment surveillance has included regular clinical reviews and interval contrast‐enhanced CT of the neck and chest. She has experienced progressive improvement in dyspnea and hoarseness, with return to baseline functional status at her most recent visit. Interval imaging at 21 months demonstrated no measurable residual or recurrent disease on contrast CT. Serum thyroglobulin was not informative for surveillance in this anaplastic tumor and was not used. She continues regular oncologic follow‐up.

## 3 Discussion

ATC carries an extremely poor prognosis, with a median survival of only a few months. Large series and national cohorts report median overall survival of ~2–6 months, and 1‐year survival around 10%–20% [3, 8]. For example, in a Philippine case series (*n* = 15), median survival was 3 months, and > 90% died within 1 year [5]. In a Dutch registry study (*n* = 812), median survival was 2.2 months (1‐year survival ~12%) [7]. Thus, survival beyond 1 year is quite uncommon; only a small minority (≈10%–20%) reach 12 months, and long‐term survivors (beyond 2–3 years) are very rare [5, 8]. By contrast, our patient's 21‐month survival is highly unusual, suggesting that selected cases can achieve prolonged survival when aggressive therapy is applied. Previous reports have noted that patients who are younger, have smaller tumors and no distant metastases, and undergo complete resection can occasionally attain long‐term remission [6, 14].

Making the diagnosis of ATC can be challenging. It may mimic other thyroid malignancies [2, 15]. Poorly differentiated thyroid carcinoma (PDTC) is a tumor type with intermediate features and somewhat better prognosis; PDTC often shows some residual thyroid architecture or thyroglobulin production, whereas ATC loses thyroid differentiation entirely [15, 16]. Primary thyroid lymphoma (usually diffuse large B‐cell type) can present as a rapidly growing neck mass in older patients, but it usually has lymphoid cytology and a different immunoprofile [17]. Metastatic cancer to the thyroid (e.g., from lung or kidney) is another mimic [18]. In practice, cytologic and histologic evaluation is needed: ATC characteristically shows undifferentiated spindle, giant, or squamoid cells with necrosis [2, 3, 15]. Notably, older classification systems even included some “small cell” ATC, which was later recognized to include many lymphomas and was dropped [19]. In this patient, the cytology was straightforwardly consistent with ATC, and there were no immunohistochemical markers of alternative diagnoses. The final surgical pathology confirmed ATC.

Multimodal therapy is the mainstay for ATC. With complete resection when feasible, high‐dose external beam radiotherapy to the neck and systemic therapy tailored by molecular profile offers the best chance to prolong survival [9]. One review noted that if the tumor is intrathyroidal, aggressive resection “may provide the best chance to prolong survival” [6]. However, most patients are not resectable due to local invasion or metastases [4]. However, at our centre, radiotherapy and approved targeted agents were not available, and molecular profiling was not performed; therefore, our approach relied on neoadjuvant and adjuvant systemic chemotherapy combined with a near‐total lobectomy as an adapted, resource‐sensitive strategy. Adjuvant radiation therapy (usually high‐dose external beam) is also recommended and has been associated with improved outcomes [14, 20], though resource constraints meant radiation was not fully administered here.

Multimodal therapy is the mainstay for ATC. Complete resection when feasible, high‐dose external beam radiotherapy to the neck, and systemic therapy tailored by molecular profile currently offer the best chance to prolong survival [9]. One review noted that if the tumor is intrathyroidal, aggressive resection “may provide the best chance to prolong survival” [6], although most patients are unresectable due to local invasion or metastases [4]. Current guidelines also recommend molecular testing to guide targeted therapy, including BRAF/MEK inhibitors for BRAF V600E–mutant tumors [9]. In our centre, however, radiotherapy, molecular profiling, and approved targeted agents were not available; therefore, our approach relied on neoadjuvant and adjuvant systemic chemotherapy combined with a near‐total lobectomy as an adapted, resource‐sensitive strategy. Adjuvant radiation therapy (usually high‐dose external beam) is also recommended and has been associated with improved outcomes [14, 20], but resource constraints prevented its administration in this case.

Chemotherapy alone has historically had limited efficacy with low response rates [10, 20]. In recent years, targeted agents have changed the landscape. For BRAF V600E‐mutated ATC, the combination of dabrafenib plus trametinib yields response rates > 50% and has regulatory approval [6, 12]. In the Phase II ROAR trial update, this regimen showed a 69% objective response and a median survival of ~14 months in BRAF‐mutant ATC [21]. Immunotherapy (checkpoint inhibitors) is under active study; early data suggest possible benefit in some patients [11, 13]. In practice, access to these novel therapies is limited in many settings. Our patient did not receive targeted or immune therapy, as her tumor's mutational status was unknown, and such agents are not readily available in Ethiopia.

Resource limitations substantially affected management. In many low‐income settings, advanced techniques like IMRT or combination targeted drugs are not options [2, 9]. In the Philippine series, for example, none of the patients received chemotherapy, and only a few could complete radiotherapy [5]. Similarly, our patient's management depended on conventional surgery and systemic chemotherapy, as novel targeted agents and radiotherapy were not available. Because she presented with significant tracheal compromise, neoadjuvant systemic chemotherapy was prioritized to achieve cytoreduction and facilitate a safer resection. The absence of radiotherapy and targeted therapies reflects local resource constraints and limits direct comparison with series where these modalities are routinely incorporated. The fact that she achieved 21‐month remission under these conditions highlights the impact of aggressive combined modality treatment, even if some components (like full‐dose radiation) must be abbreviated. We used clinical assessment and interval contrast‐enhanced CT to define remission; biochemical markers such as thyroglobulin have limited sensitivity in ATC and were not relied upon, which we acknowledge as a limitation of surveillance strategies in this disease.

Notably, several prognostic factors in ATC have been identified. Favorable factors include younger age (< 60–65 years), smaller tumor size, no distant metastases at diagnosis, and comprehensive treatment [4, 6, 7]. In the Dutch study, multivariable analysis showed that triple therapy (surgery + radiotherapy + chemotherapy), age < 65, and absence of metastases were independently associated with longer survival [7]. Our patient had no distant metastases and received multi‐modality therapy, which likely contributed to her unusually long survival [14]. This case underscores that even in ATC, a subset of patients with limited disease burden can achieve extended survival with aggressive care [2, 4, 8]. We therefore do not claim improved survival from the intervention itself, but rather interpret the 21‐month outcome as consistent with the favorable prognostic profile in this individual patient. We also emphasize that this is a single case report subject to selection bias and cannot demonstrate treatment efficacy; broader studies are needed to define which patients may benefit from similar approaches.

## 4 Conclusion

This case illustrates that ATC, though generally lethal, can occasionally have a protracted course when managed aggressively. Key lessons include the importance of early diagnosis and an aggressive, multidisciplinary approach combining surgery, chemotherapy, and radiotherapy whenever feasible. Differentiating ATC from other thyroid malignancies requires careful histopathology, and clinicians should maintain a high index of suspicion for ATC in rapidly growing thyroid masses. In resource‐limited settings, maximizing available modalities can still yield meaningful extensions of life. Ultimately, this case highlights that multidisciplinary intervention can significantly affect prognosis, and it reinforces the need for better access to advanced therapies and continued research into prognostic markers and novel treatments for ATC. While this single case does not establish generalisability, it suggests that selected patients with locally advanced, nonmetastatic ATC may achieve prolonged survival with aggressive multimodal therapy even in resource‐limited settings, underscoring the need for further study.

## Author Contributions

**Dereje G. Andargie:** conceptualization, writing – original draft. **Chernet T. Mengistie:** visualization, writing – original draft. **Biruk T. Mengistie:** visualization, writing – review and editing. **Solomon Hunegnaw:** data curation, resources. **Anduamalak Bazezew:** data curation, resources. **Kristin Long:** supervision, writing – review and editing. **Raj Petersson:** supervision, writing – review and editing.

## Funding

The authors have nothing to report.

## Ethics Statement

IRB review and approval were waived for this case report.

## Consent

Written permission for publication of the clinical details and accompanying images was obtained; the signed consent form is held by the corresponding author and can be made available to the Editor on request.

## Conflicts of Interest

The authors declare no conflicts of interest.

## Data Availability

The data underlying the results presented in this work are available within the manuscript.
